# Hydrogen–nitrogen plasma assisted synthesis of titanium dioxide with enhanced performance as anode for sodium ion batteries

**DOI:** 10.1038/s41598-020-68838-x

**Published:** 2020-07-16

**Authors:** Hongmei Wang, Jie Xiong, Xing Cheng, Ge Chen, Thomas Kups, Dong Wang, Peter Schaaf

**Affiliations:** 1 Fachgebiet Werkstoffe der Elektrotechnik, Institut für Werkstofftechnik und Institut für Mikro-Und Nanotechnologien MacroNano®, TU Ilmenau, Gustav-Kirchhoff-Str. 5, 98693 Ilmenau, Germany; 2 Beijing Key Laboratory for Green Catalysis and Separation, College of Environmental and Energy Engineering, Beijing University of Technology, 100124 Beijing, People’s Republic of China

**Keywords:** Batteries, Batteries

## Abstract

Sodium ion batteries are considered as one of the most promising energy storage devices as lithium ion batteries due to the natural abundance of sodium. TiO_2_ is very popular as anode materials for both lithium and sodium ion batteries because of the nontoxicity, safety and great stabilities. However, the low electronic conductivities and inferior sodium ion diffusion make it becoming a great challenge to develop advanced TiO_2_ anodes. Doping heteroatoms and incorporation of defects are believed to be great ways to improve the electrochemical performance of TiO_2_ anodes. In this work, commercial TiO_2_ (P25) nanoparticles was modified by hydrogen and nitrogen high-power plasma resulting in a disordered surface layer formation and nitrogen doping as well. The electrochemical performances of the samples as anode materials for sodium ion batteries was measured and the results indicated that after the hydrogen–nitrogen plasma treatment, H–N-TiO_2_ electrode shows a 43.5% of capacity higher than the P-TiO_2_ after 400 cycles long-term discharge/charge process, and the samples show a good long cycling stability as well, the Coulombic efficiencies of all samples are nearly 99% after 50 cycles which could be sustained to the end of long cycling. In addition, hydrogen–nitrogen plasma treated TiO_2_ electrode reached the stable high Coulombic efficiency earlier than the pristine material. High resolution TEM images and XPS results indicate that there is a disordered surface layer formed after the plasma treatment, by which defects (oxygen vacancies) and N-doping are also introduced into the crystalline structure. All these contribute to the enhancement of the electrochemical performance.

## Introduction

Rechargeable sodium ion batteries (SIBs) have been considered as the competitive alternative to lithium ion batteries because of some special merits, such as environment friendly, low cost and especially abundant alkali element widely distributed on earth^1–3^. However, there is a big challenge that the ion radius of Na ions are ~ 70% larger than that of Li ions, so finding proper electrode materials which could provide big interstitial space to accommodate sodium ions and allow reversible and rapid ion insertion/extraction^3^ is a challenging topic at the moment. Recently, many efforts have been made to explore advanced anode materials for sodium ion batteries. Firstly, carbonaceous material is an important choice which is very cheap however carbonaceous materials show undesired properties with low capacities and/or poor cycle performance^4^. Secondly, Ti-based materials is a promising anode materials for sodium ion batteries, including Na_2_Ti_3_O_7_, Na_0.66_[Li_0.22_Ti_0.78_]O_2_, Li_4_Ti_5_O_12_, and titanium dioxide (TiO_2_)^5^. Moreover, other materials based on alloying reactions (such as Sn, Sb, P and their compounds e.g. inter-metallics, oxides, sulfides, and phosphides)^6–10^ and conversion reactions (oxides and sulfides, e.g. Fe_2_O_3_, Fe_3_O_4_, FeOOH, MoO_3_, CuO, Mn_3_O_4_, NiCo_2_O_4_ and MoS_2_)^11, 12^ attract some attentions as well. In addition, organic compounds are also potential anode materials for sodium ion batteries^13–15^.

Titanium dioxide (TiO_2_) with various polymorphs like anatase^16^, rutile^17^, amorphous^18^ TiO_2_ and TiO_2_-B^19^ as anode materials have been investigated, and it is generally considered as a promising energy storage material because of the low cost, environmental friendliness, intrinsic safety, abundant resources, high power density and long cycle life^20, 21^. Nevertheless, the big limitation of using TiO_2_ as anode for sodium ion batteries is its inherent low electrical conductivity. In order to solve this problem, firstly, surface coating could be a good method to improve the TiO_2_ conductivity, especially introducing carbon additives to enhance the electrical conductivity of TiO_2_ anodes, like amorphous carbon, CNTs and graphene have been widely studied as conductive agents^22–27^. Secondly, designing nanosized TiO_2_, for example nanotubes^28^, nanoparticles^29^, nanorods^28^ and nanofiber^30^, petal-like TiO_2_^20^, is also a good way because the nanosized structure could effectively enlarge the active area of the material and shorten the ion diffusion path during electrochemical processes. He et al.^31^ reported a hierarchical rod-in-tube TiO_2_ with a uniform carbon coating as the anode material for sodium-ion batteries by a facile solvothermal method. The author claimed that this unique structure consists of a tunable nanorod core, interstitial hollow spaces, and a functional nanotube shell assembled from two-dimensional nanosheets. What’s more, heteroatom doping like N, S, B, P or Nb^32–39^, which could substantially increase the electronic conductivity and the specific capacities for the sodium storage. A nitrogen-doped carbon layer coated yolk-like TiO_2_ electrode could offer a superior high capacity of 115.9 mAh/g^1^ at 20 C (6700 mA/g)^27^. It is clear that the key factor to improve the electrochemical performance of TiO_2_ is to combine strategies of shortening sodium-ion diffusion distance and improving electronic conductivity. In addition, the produced oxygen vacancies or trivalent titanium species can be formed to improve the electrical conductivity as well^3, 32, 40^. Wang and co-workers proposed a novel and facile N_2_ plasma assisted annealing strategy, by which nitrogen heteroatoms and rich oxygen vacancies are incorporated into the TiO_2_ crystal simultaneously, leading to highly enhanced electronic conductivity^40^. In addition, previous work of our group has already shown that plasma treatment is a promising method to enhance the performance of TiO_2_ as anode material. A hydrogen plasma treated TiO_2_ as anode for lithium ion batteries shows that the treated black TiO_2_ shows great improvement for fast lithium storage^41^. Furthermore, another work shows that nitrogen plasma treated TiO_2_ with a disordered surface layer and nitrogen doping shows an enhanced performance for sodium ion batteries^32^.

Here, we developed a combination of hydrogen and nitrogen plasma assisted strategy to synthesize nitrogen doping and defect-rich (oxygen vacancies) TiO_2_ electrode that demonstrated an enhanced performance as anode material of sodium ion batteries. In our research, there are three electrodes which are tested, pristine commercial TiO_2_ as control sample (named as P-TiO_2_), high-power hydrogen plasma treated TiO_2_ (named as H-TiO_2_) and high-power nitrogen plasma treated H-TiO_2_ (named as H–N-TiO_2_). When used as anode materials, the H–N-TiO_2_ shows the best sodium storage performance, and both H-N-TiO_2_ and H-TiO_2_ demonstrated much higher specific capacities than pristine TiO_2_, and also the long-term performance results are promising. The high-resolution images of transmission electron microscopy shown that there is a disordered surface layer was formed after the plasma treated materials, no matter the hydrogen or nitrogen plasma process. For this improvement of the electrodes, we could attribute it to the disordered surface layer, the oxygen vacancies and the nitrogen doping, and all of them play significant roles in enhancing the electrochemical sodium storage performance.

## Experimental section

### Sample preparation

The sample preparation process is similar with our previous work^32, 42^. Commercial TiO_2_ (P25) nanoparticles were purchased from Sigma-Adlrich and used without further purification. 0.15 g TiO_2_ nanoparticles were dispersed in 30 ml ethanol with ultrasonic support and drop-casted onto a 6-inch Si wafer. The drop-casting process was repeated several times to achieve TiO_2_ mass loading of about 0.8 mg cm^−2^ (sample mass loading should be no more than 1.5 mg cm^−2^ to avoid inhomogeneous plasma treatment). Before each drop, the previous dropped ethanol must be volatilized totally so that a homogenous distribution of the nanoparticles on the silicon wafer can be obtained.

The drop-casted wafer was transferred into a chamber for the plasma treatment, and the instrument of inductively coupled plasma enhanced chemical vapor deposition (ICP-CVD, Plasmalab 100, Oxford) was used. Before the plasma treatment process, a preconditioning process takes place at 300 °C to ensure the quality of plasma treatment. Then H_2_ plasma treatment was performed at 300 °C for 30 min, the ICP power was 3000 W, chamber pressure was 3.52–3.76 Pa, and H_2_ flow rate was 50 sccm. Afterwards, N_2_ plasma treatment was performed at 300 °C for 30 min, the ICP power was 3000 W, the chamber pressure was 3.52–3.76 Pa, and N_2_ flow rate was 30 sccm. After all plasma treatment, H-TiO_2_ and H–N-TiO_2_ nanoparticles were obtained and scratched from the Si wafer for further investigations and application measurements.

### Characterization

The crystal structure of the TiO_2_ nanoparticles was characterized by X-ray diffraction (XRD, SIEMENS D5000) with Cu Kα radiation. The sample was scanned from 2θ = 10°–80° at a rate of 0.03° s^-1^ in Bragg–Brentano geometry. Then, transmission electron microscopy (TEM, Tecnai F20) was used to characterize the nanoparticle morphology and microstructure. Ultraviolet–visible (UV–Vis) absorption spectra were measured by using a Cary 5000 UV–Vis-NIR. Finally, the samples were analyzed by XPS using a spectrometer (PHI Quantera SXM) with monochromatized Al-Kα radiation.

### Electrochemical experiments

The electrochemical performances of the materials used as anode for sodium-ion batteries were tested via 2032 coin half-cells, which were assembled in an argon-filled glove box, where both the moisture and oxygen contents were less than 0.5 ppm. The working electrode was immersed in a mixture, which consist of active material, super carbon black and polyvinylidene in ratio of 70: 15: 15, toward forming a homogeneous slurry in N-methyl-pyrrolidone (NMP). Then, the slurry was pasted onto stainless steel foil with a sample loading of about 1.8 mg cm^−2^. The handled electrode was dried for 12 h at 120 °C under vacuum. Glass fiber (GF/D, Whatman) and pure sodium foil (Aldrich) were respectively used as a separator and the counter electrode. The electrolyte was 1 M NaPF_6_ in a mixture of ethylene carbonate and diethyl carbonate, in a ratio of 50:50. For the galvanostatic measurement, a battery tester (Neware, Shenzhen, China) was used. Cyclic voltammetry (CV) was applied in a potential range from 3.0 to 0.01 V at a scan rate of 0.5 mV s^−1^ by a potentiostat (VMP3, BioLogics, France). Furthermore, the alternating current (AC) impedance of the samples were determined by a same potentiostat and the impedance spectra, which were acquired via a sine wave with an amplitude of 5.0 mV in a frequency range from 100 kHz to 0.01 Hz after discharging/charging for 5 cycles at a current density of 1 A g^−1^.

## Results and discussion

**Figure 1:**
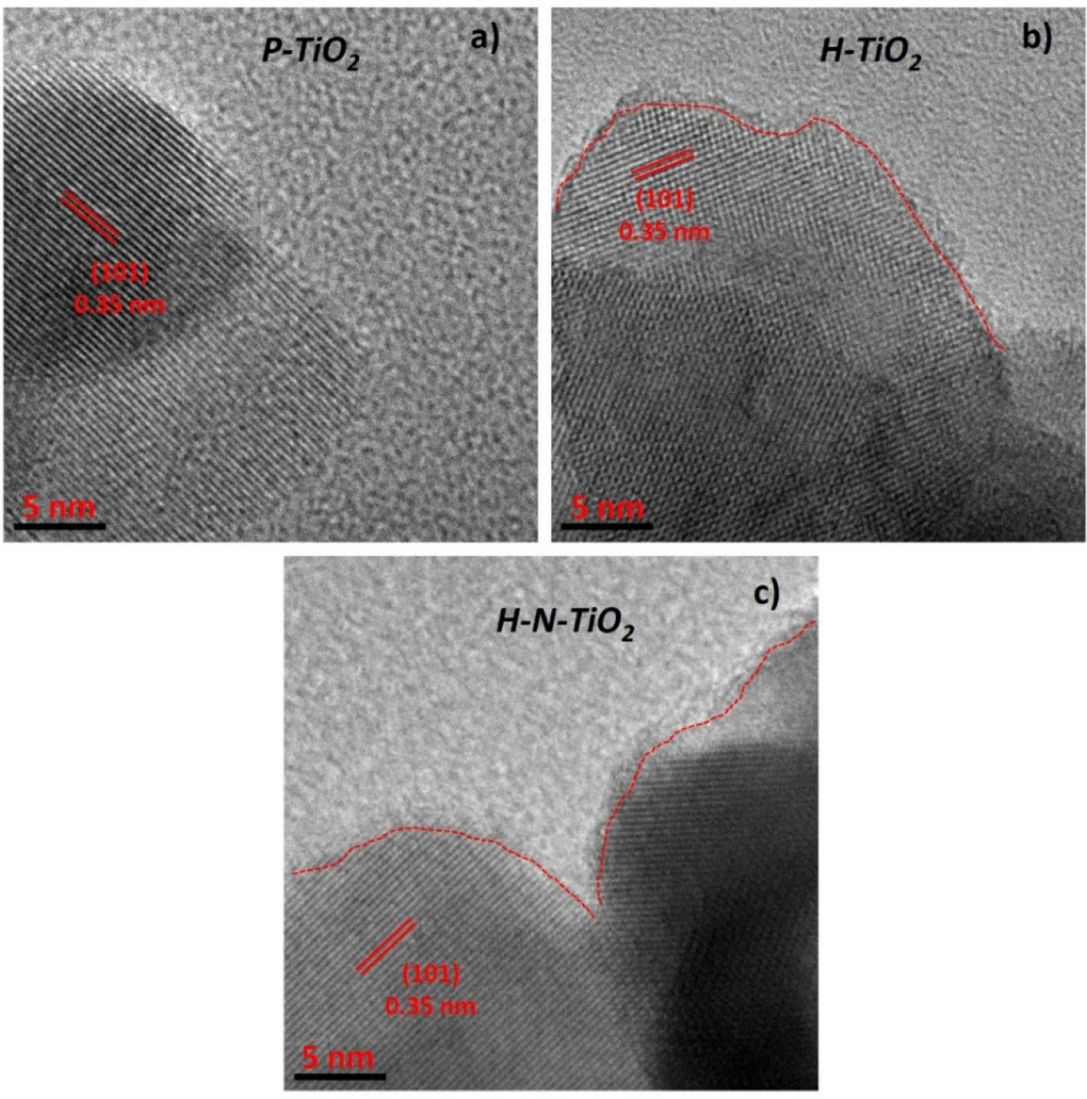
High resolution TEM images of (**a**) the pristine (P-TiO_2_) nanoparticles, (**b**) the nanoparticles treated with H_2_ plasma (H-TiO_2_), and (c) the nanoparticles treated with both H_2_ and N_2_ plasma (H–N-TiO_2_).

Figure 1 shows the high-resolution TEM images of the pristine TiO_2_ nanoparticles and the TiO_2_ nanoparticles after plasma treatments. We could see that all TiO_2_ nanoparticles were highly crystallized, and the interplane spacing of ordered lattices is measured to be closed to 0.35 nm, which could be attributed to (101) planes of the anatase crystal phase. Moreover, we could find that the crystal lines of plasma treated material (H-TiO_2_ and H–N-TiO_2_) do not arrange clearly compared with the pristine TiO_2_, and there are some crossovers of the fringes which may be a partially dislocation or defects in the TiO_2_. What’s more, a disordered layer was found in Fig. 1b, c, and the thickness of the disordered surface layer is about 1.5 nm, we think this is due to the high-power plasma treatment processes. According to previous report^43^, the thickness of the disordered surface layer could be increase upon the plasma treatment time, after 20 min treatment, the thickness reaches to around 1.5–2.2 nm and the Electron paramagnetic resonance (EPR) result indicated that hydrogen plasma treatment is very effective to produce Ti^3+^ species and oxygen vacancies, which resulted in the disordered surface layer formation.

**Figure 2:**
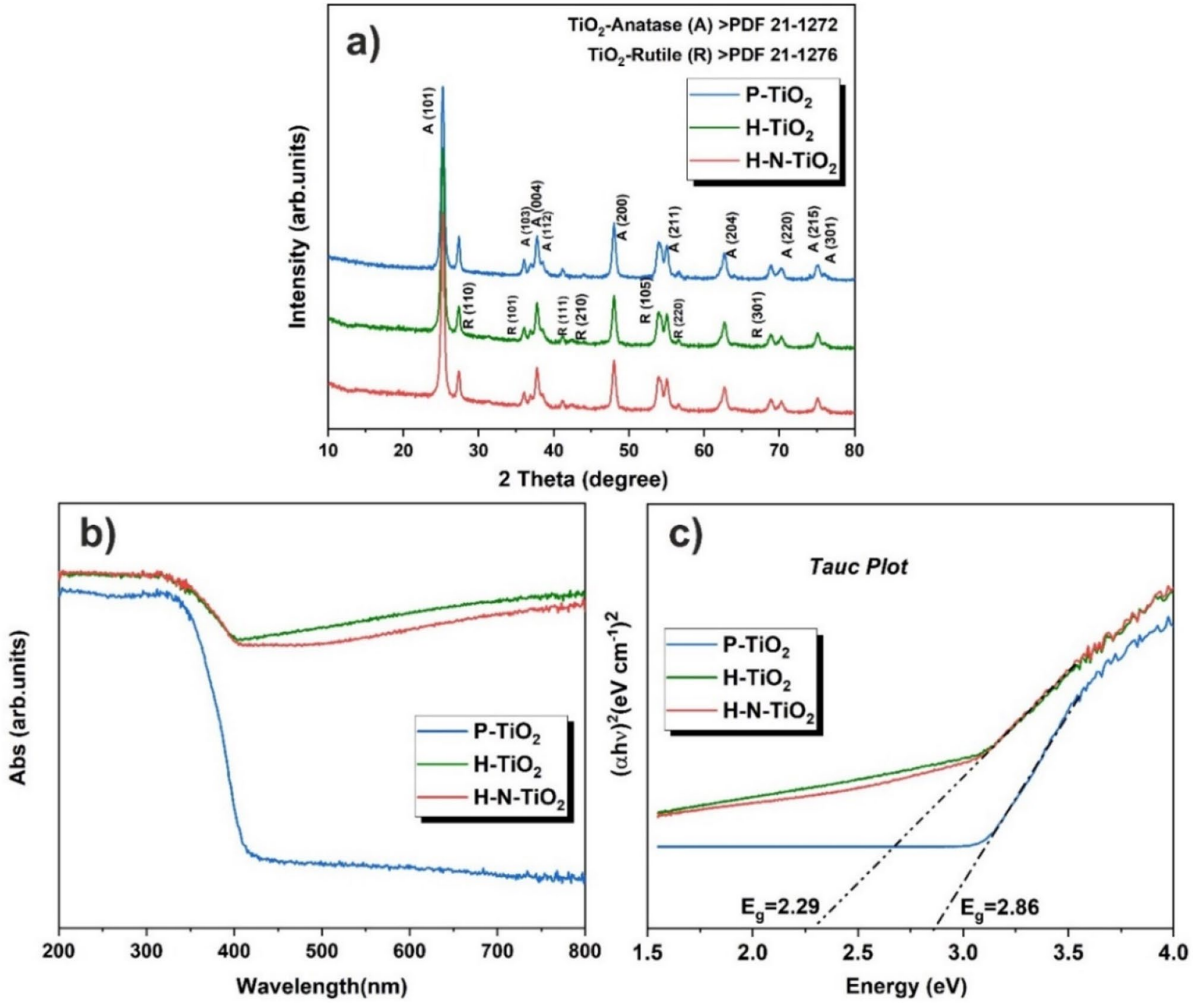
(**a**) X-ray diffraction patterns of P-TiO_2_, H-TiO_2_ and H–N-TiO_2_; (**b**) UV–Vis absorption spectra of P-TiO_2_, H-TiO_2_ and H–N-TiO_2_ and (**c**) Tauc plots to obtain the band gap.

The XRD patterns of the samples are shown in Fig. 2a, from which we could see there are two different phases of TiO_2_, anatase and rutile. The peaks appearing at 2θ = 25.4°, 36.0°, 37.8°, 38.5°, 48.0°, 55.0°, 62.7°, 70.2°, 75.1° and 76.0° correspond to the (101), (103), (004), (112), (200), (211), (204), (220), (215) and (301) diffractions of anatase TiO_2_ (PDF No. 21-1272), respectively. And the other peaks which located at 2θ = 27.3°, 36.0°, 41.2°, 42.5°, 54.0°, 56.6° and 68.8° could be attributed to the (110), (101), (111), (210), (105), (220) and (301) diffractions of rutile TiO_2_ (PDF No. 21-1276), respectively. The intensities of the peaks in the P-TiO_2_ are slightly higher than those in the plasma treated materials. Except these small differences, all the diffraction peaks are almost the same. In addition, the peaks of nitrides were not clearly observed in the patterns, probably due to the low amount of doping contents and also the homogeneous distribution of N in the sample^44, 45^. The UV–Vis absorbance of the samples was investigated and Tauc plots were calculated to determine the band gap energy of the materials. As shown in Fig. 2b, P-TiO_2_ shows the absorption edge at around 427 nm, while after plasma treatment, the absorption edge shifted to ~ 685 nm for both H-TiO_2_ and H–N-TiO_2_. The strongly enhanced absorption of the plasma treated materials in the region of visible light indicates that the high-power plasma treatment can result in a reduction effect and oxygen vacancies are formed on the materials surface^41, 43^. Tauc plots (Fig. 2c) clarifies the decline of band gap energy of the materials from 2.86 to 2.29 eV due to the hydrogenation process^41^ and nitrogen doping^46–48^.

**Figure 3:**
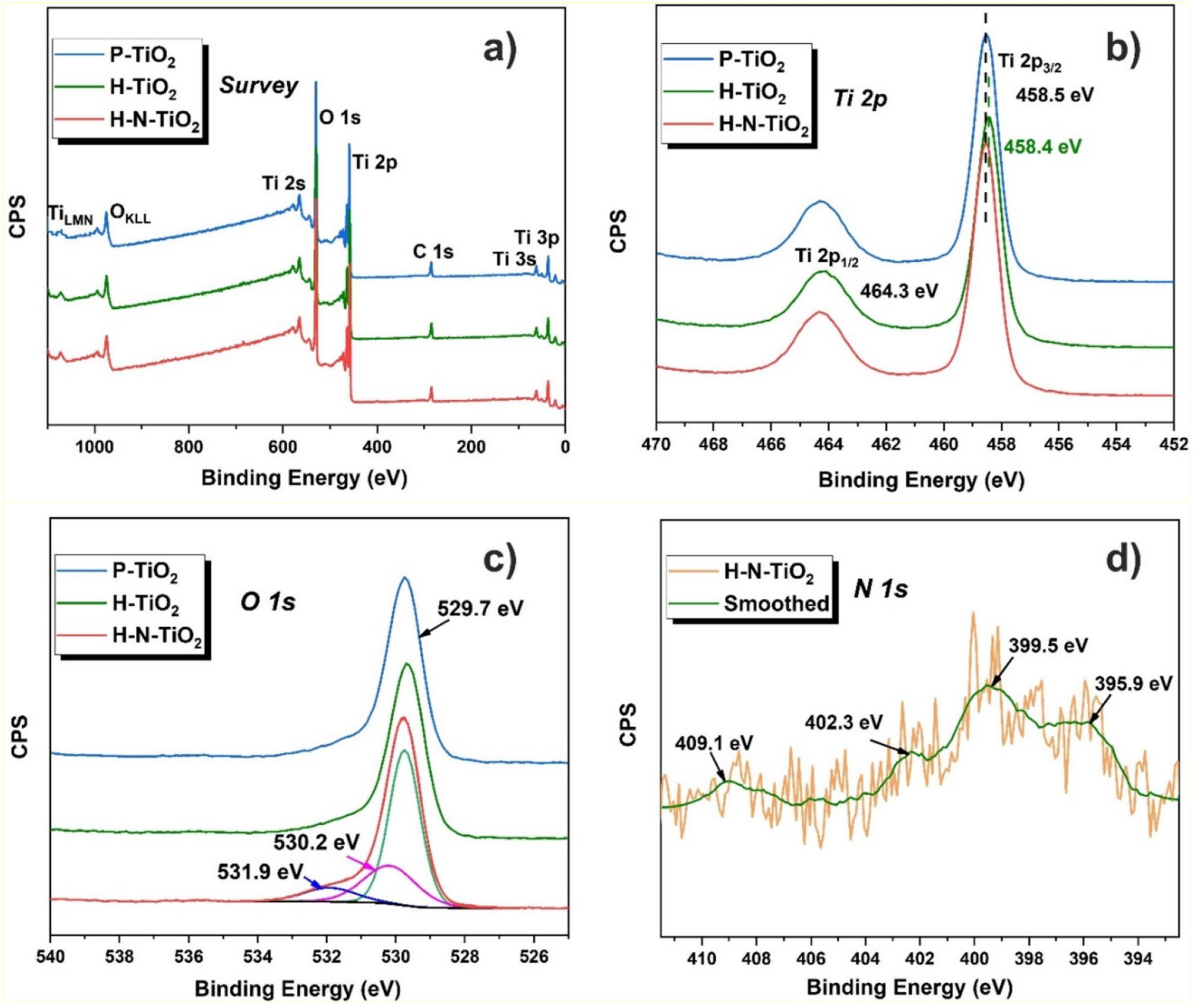
(**a**) XPS survey spectra of TiO_2_ electrodes; (**b**) Ti 2p spectra of TiO_2_ electrodes; (**c**) O 1s spectra of TiO_2_ electrodes; (**d**) N 1s spectrum of H–N-TiO_2_.

XPS was used to investigate the surface chemical structure details of the obtained samples (shown in Fig. 3), Ti, O and C elements could be observed from the survey spectra in Fig. 3a. For the further analysis of the chemical structure of the investigated TiO_2_ samples, high-resolution XPS spectra of all elements are processed. Figure 3b shows the high-resolution spectra of Ti 2p, from the spectra we could see there are two typical peaks which centered at 464.3 and 458.5 eV for all samples. They could be attributed to the Ti 2p_1/2_ and Ti 2p_3/2_, respectively, which represent the characteristic peaks of Ti^4+^. What’s more, compared to the other two samples, the Ti 2p_3/2_ peak of H-TiO_2_ shifted to the lower binding energy slightly, but the peak difference is so weak and we would say that the change of chemical environment of titanium is not obvious after high-power plasma treatment.

Figure 3c shows the XPS spectra of O 1s, there is one main peak located at 529.7 eV for the pristine and hydrogen treated materials, which is attributed to bulk oxygen O^2−^. For H–N-TiO_2_, two new characteristic peaks appear after plasma treatment, which are centered at 530.2 and 531.9 eV, corresponding to the chemisorbed oxygen of the surface hydroxyl, absorbed H_2_O, absorbed O_2_ or the surface contamination^49^. Further, the peak centered at 531.9 eV is attributed to the presence of Ti–O–N bonds^49, 50^, which indicates the formation of Ti–O–N bonds after the hydrogen and nitrogen plasma treatment.

In addition, the high-resolution XPS spectrum of N 1s of H–N-TiO_2_ is displayed in Fig. 3d. In accordance with a previous report^32^, the 30-min nitrogen plasma treatment can result in about 0.5 at% of nitrogen doping in the material. There are four characteristic peaks, which are located at 395.9, 399.5, 402.3 and 409.1 eV, respectively. Firstly, according to some reports^51, 52^ the peak of N 1s located at 395.9 eV can be attributed to O–Ti–N linkages, which comes from the doping of N atoms into the TiO_2_ lattice. Another peak at 399.4 eV can be attributed to interstitial N^49^. Furthermore, two other peaks at 402.3 and 409.8 eV belong to NO or NO_2_ type species which usually appear at binding energies higher than 400 eV^50^. From the above observations, the chemical states of the nitrogen doped TiO_2_ exist with the form of N–Ti–O and Ti–O–N^46^. Many research works have been reported that the role of N species on the electrical conductivity of TiO_2_ is mainly related to the decrement of the band gap, because nitrogen doping can elevate the valence band maximum^53^.

## Electrochemical performances of sodium ion batteries

**Figure 4:**
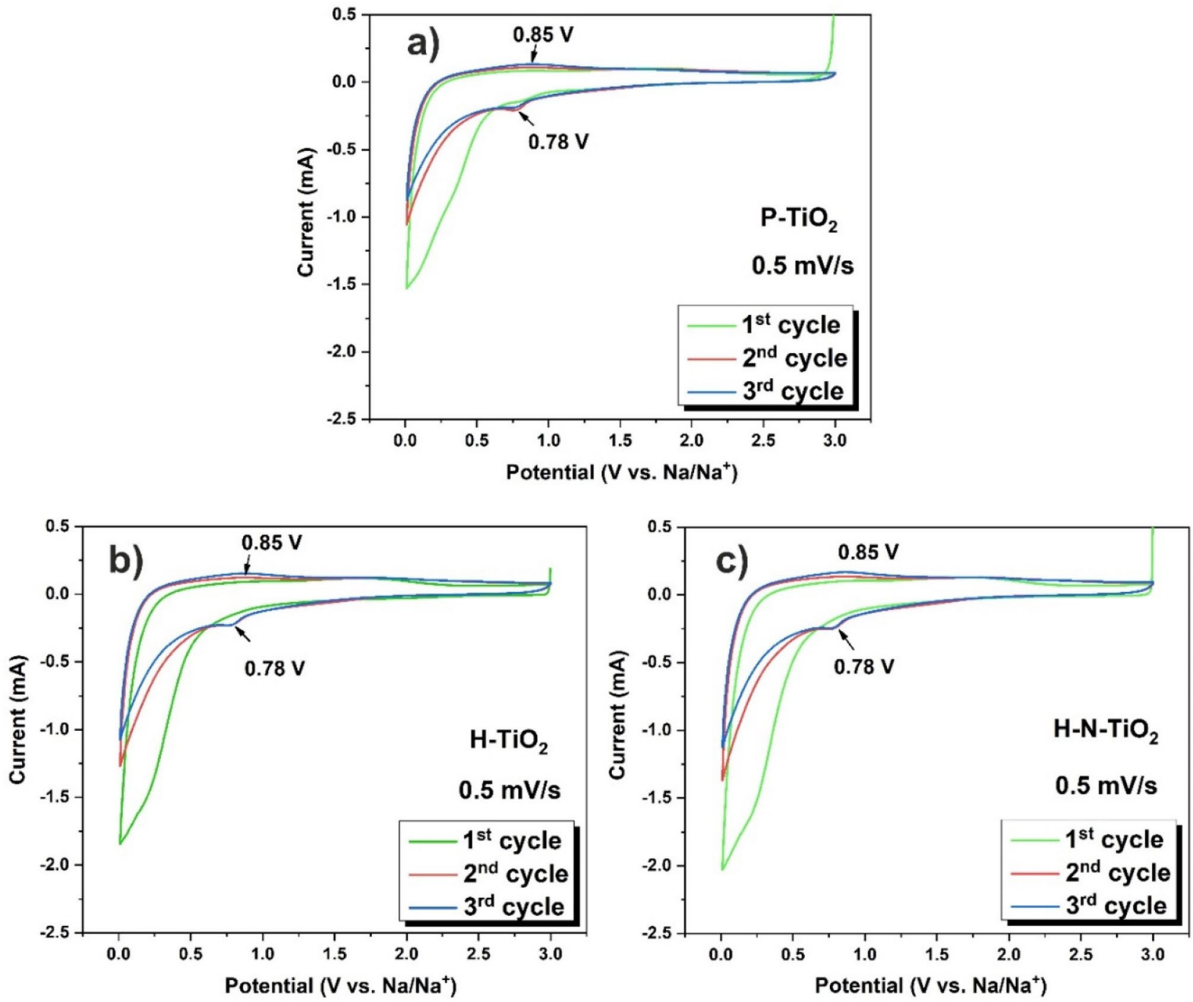
(**a**) CV curves of TiO_2_ electrodes measured at a scan rate of 0.5 mV s^-1^ for 3 cycles, (**a**) P-TiO_2_; (**b**) H-TiO_2_; (**c**) H–N-TiO_2_.

The electrochemical performances of the TiO_2_ employed as anode materials for sodium-ion batteries were evaluated in coin half-cells using sodium metal as the counter electrode. Firstly, the cycle voltammetry (CV) plots which were evaluated at a scan rate of 0.5 mV/s at a voltage range of 0.01–3.0 V for 3 cycles of each sample are shown in Fig. 4. All of the TiO_2_ electrodes display the typical CV curves of TiO_2_ anode for SIBs^29, 54^. From the first cycle, irreversible broad cathode peaks at wide potential range of 0.01–0.5 V are observed for all samples (Fig. 4), which can be attributed to the irreversible sites for Na-ion insertion in the crystal lattice defects, electrolyte and other organic material decomposition and the solid electrolyte interface (SEI) layer formation^54, 55^. From the second and third cycles, there are only the peaks at around 0.78 and 0.85 V are shown in the CV profiles for all obtained samples (Fig. 4), which are corresponding to the reversible insertion/de-insertion of Na into/from the electrodes. Moreover, except the first cycle, the CV curves of all three samples overlapped well for the second and third cycles, which demonstrated good cycling stability and the high reversibility of TiO_2_ electrodes. What’s more, the peak current of the H-TiO_2_ and H–N-TiO_2_ are wider and stronger than those of the P-TiO_2_ electrode, and it means the plasma treated materials show better sodium storage performances (Fig. 4b,c).

**Figure 5:**
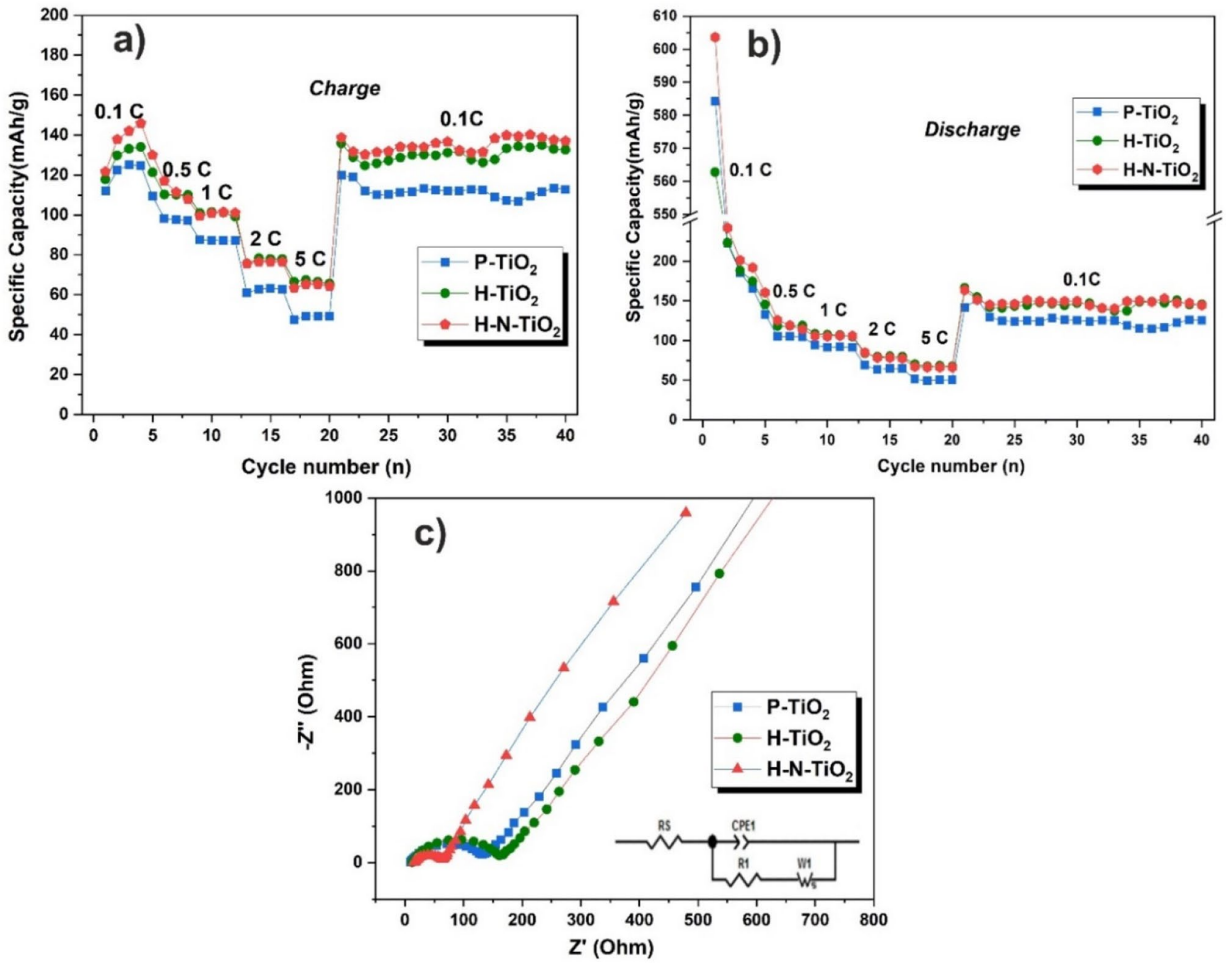
Rate performances of TiO_2_ electrodes at the current densities of 0.1, 0.5, 1.0, 2.0 and 5.0 C, (**a**) charge, (**b**) discharge; (**c**) Nyquist plots of TiO_2_ electrodes.

The rate performances of all samples are given in Fig. 5a, b, all obtained samples were measured at current densities of 0.1, 0.5, 1, 2 and 5 C for 4 cycles of every step, and then the current densities came back to 0.1 C for 10 cycles (1C = 335 mA/g). The first cycle discharge/ charge specific capacities of P-TiO_2_, H-TiO_2_ and H–N-TiO_2_ electrodes, are 584.28/112.08, 562.86/117.70 and 603.60/121.65 mAh/g, respectively. A very low coulombic efficiency for about 20% are obtained for all the materials at the first cycles. The irreversible capacities of the electrodes were mainly caused by the occurrence of the side reactions which could form a solid-electrolyte interface (SEI) layer. It is hard to say which treated material shown a better electrochemical performance, but we are sure that the plasma treatment methods have a good effect on the TiO_2_ anode materials for SIBs. In order to further understand the improved higher performance of the TiO_2_ electrodes, electrochemical impedance spectra (EIS) was evaluated in the frequency range of 100 kHz to 0.01 Hz (as shown in Fig. 5c). The spectra were fitted by Zview software using an equivalent circuit mode. Here, R_s_ is the internal resistance in the batteries and R_1_ is charge transfer resistance on the interface of electrode and electrolyte. CPE represent a constant phase element and the sloping line in the low-frequency region related to W1, which is called Warburg impendence, could be attributed to the diffusion resistance in the electrode^56^. The charge transfer resistance of the P-TiO_2_ is about 137 Ω according to the fitted result, and increased a bit for the H-TiO_2_ electrode. While the charge transfer resistance of H–N-TiO_2_ (ca. 66 Ω) is about half of the P-TiO_2_, which means that the nitrogen doping is an easy and effective way to improve the electrical conductivity and the charge transfer reactions of the TiO_2_ anode material for sodium ion batteries. From our previous work, an individual nitrogen plasma treated TiO_2_ shown the similar result^32^.

**Figure 6:**
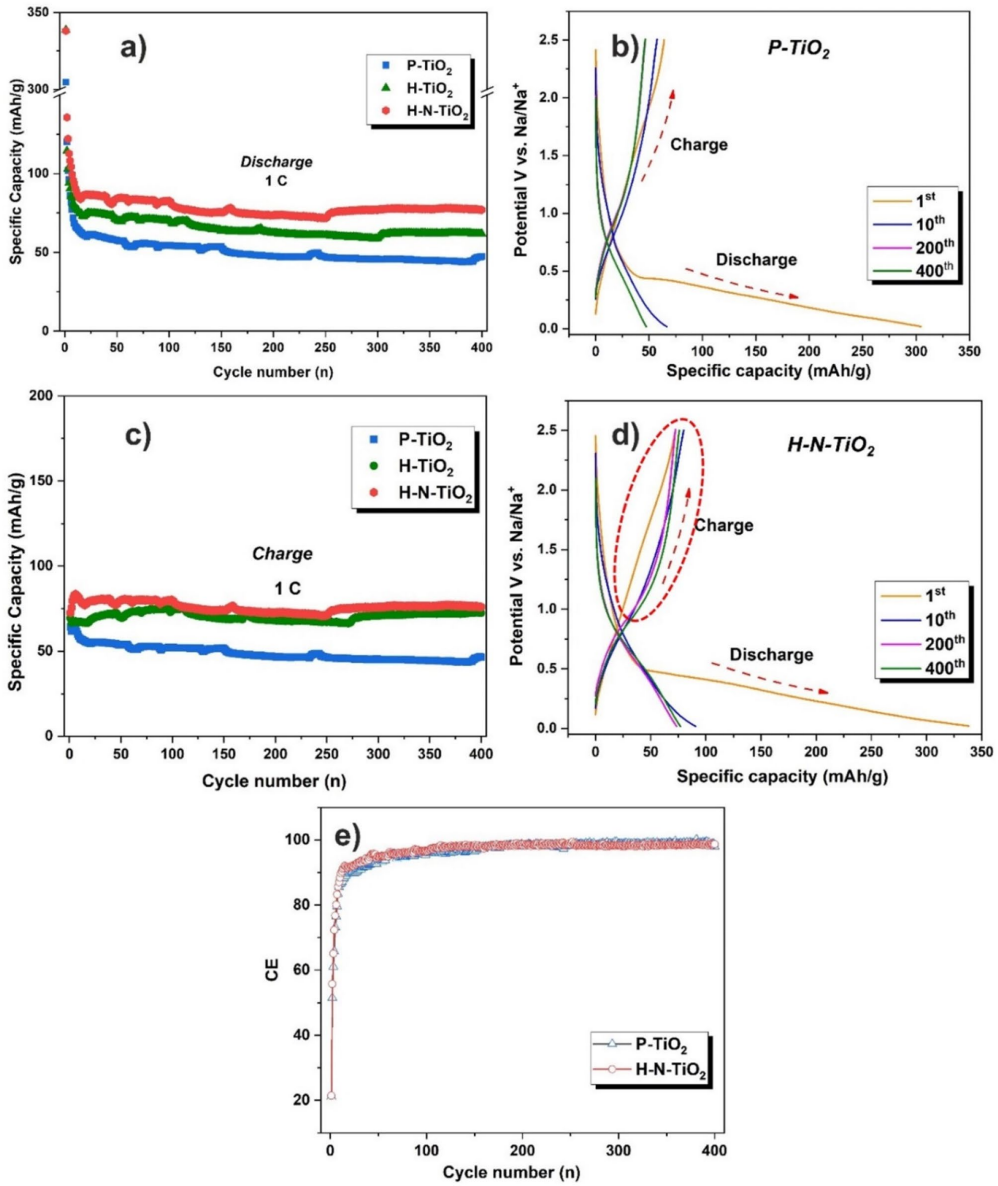
Long term cycling performances of electrodes at a current rate of 1 C (**a**) discharge; (**c**) charge; Galvanostatic discharge/charge curves of electrodes at different cycles (**b**) P-TiO_2_; (d) H–N-TiO_2_; (**e**) Coulombic efficiencies for long cycling performances of P-TiO_2_ and H–N-TiO_2_.

The long cycling stabilities of the materials were evaluated at a current rate of 1 C for 400 cycles. As is shown in Fig. 6a,c, the first discharge/charge specific capacities of P-TiO_2_, H-TiO_2_ and H–N-TiO_2_ electrode are 304.73/64.23, 325.76/69.37 and 338.07/72.54 mAh/g, respectively. It is very similar to the rate performance, all of the materials show a very low Coulombic efficiency in the first cycle, about 21%. The low Coulombic efficiency of all samples is due to the large ion radius of sodium and low theoretical capacity of TiO_2_ anodes, which can result in high irreversible capacities of anodes for sodium ion batteries. Secondly, the TiO_2_ materials we used in this work is just normal nanoparticles, we don’t have special structure to provide large amounts of active sites for the electrochemical reactions. In addition, the nitrogen concentration after plasma treatment is also very low compared with some previous reported works^37, 57^, but we could still see the big improvement of the materials as anode with the effect of plasma treatment. After about 20 cycles, the materials which were treated with hydrogen and nitrogen plasma, show higher capacities than the pristine TiO_2_. Especially for the H–N-TiO_2_, the discharge/charge capacities for the 400th cycles are 68.05/66.85 mAh/g, while the P-TiO_2_ just show specific capacities of 47.40/46.44 mAh/g, it means that H–N-TiO_2_ showing a 43.5% of capacity higher than the P-TiO_2_ after 400 long-term cycles.

Compared with our previous work^32^, hydrogen and nitrogen double plasma treatment give an improvement on the specific capacities (increased around 10% at 400th cycle) than an individual nitrogen plasma treatment, it means the hydrogenation process also makes contributions on enhancing the sodium storage performances. All of the samples show a good long cycling stability, the Coulombic efficiencies of them are nearly 99% after 50 cycles, and this could be sustained to the end of long cycling (Fig. 6e). In addition, there are some fluctuations of the capacity upon the further cycling for all samples. The reason is not clear. However, Wu^58^ and co-workers reported that during the reaction of the large size of sodium ions with the electrodes, more electrolyte penetrates from the surface of electrode into the bulk and then more active materials are participated into the reaction with the increasing of the cycling numbers; also, some other reported works^59, 60^ claim that the deposition of transition metal compounds on the anode electrode would induce the chemical degradation of SEI film. When the accumulation of produced metallic titanium reached to a certain amount, metallic titanium may catalyze and accelerate the decomposition of SEI film with the electrolyte, which could cause a sudden rise of specific capacity of electrodes.

**Figure 7:**
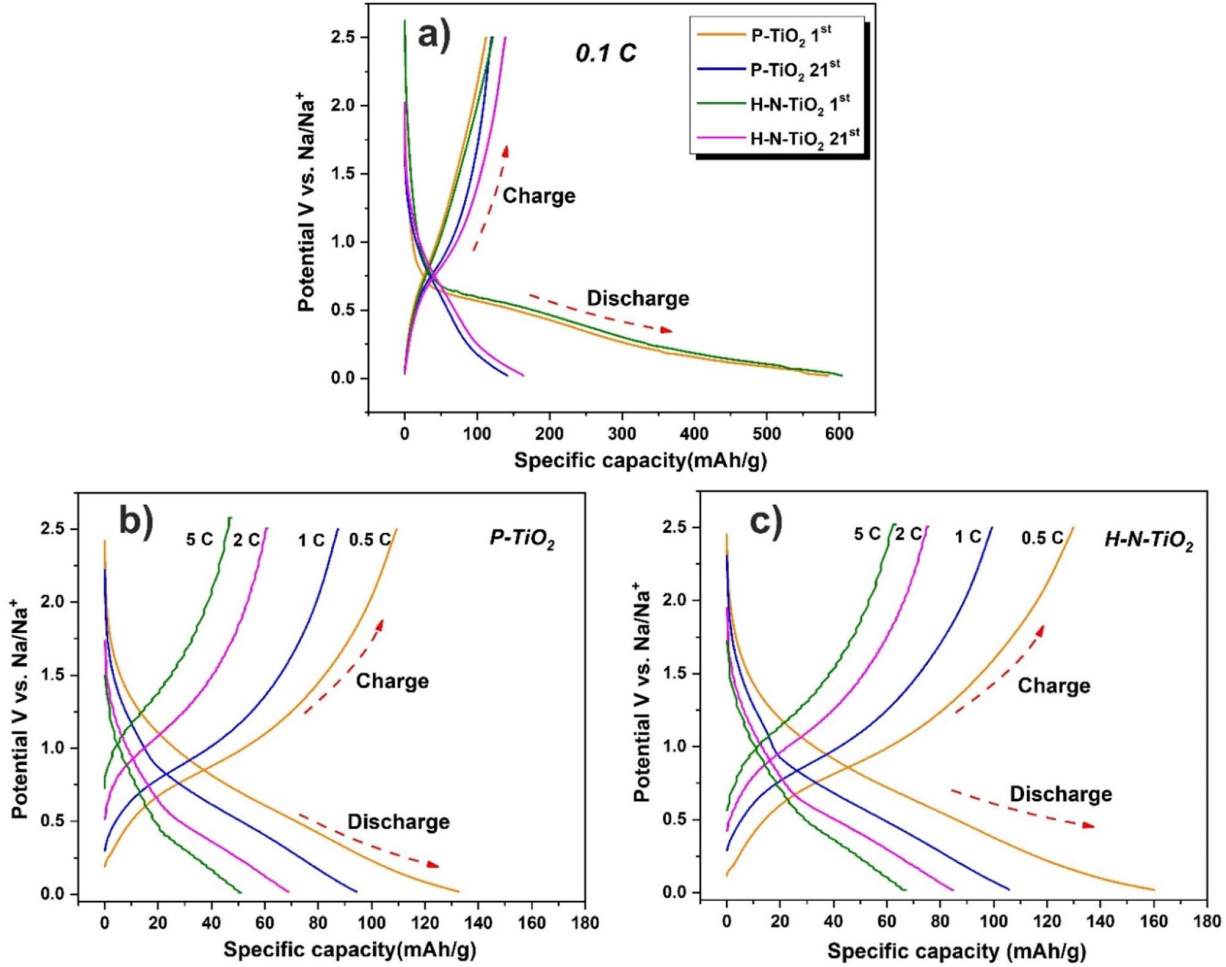
(**a**) Galvanostatic discharge/charge curves of P-TiO_2_ and H–N-TiO_2_ electrodes at current density of 0.1 C. Galvanostatic discharge/charge curves at different current densities (**b**) P-TiO_2_, (**c**) H–N-TiO_2_.

Because the different performances of the P-TiO_2_ and H–N-TiO_2_ during the long-term cycling process, so we want to get some more details from the long-term performance galvanostatic discharge and charge curves, as shown in Fig. 6b,d. The first cycle of these two materials are very similar for the charge and discharge process. But after several cycles, we could see that something is different for the charge curves. The H–N-TiO_2_ shown a slow potential increase from ~ 1.0 to 2.0 V (as shown by the red circle in Fig. 6d), it may cause by the amorphous surface layer which is good for the sodium ions transport in the material and also the nitrogen doping could greatly increase the conductivity of the TiO_2_ materials. Figure 7a is the 1st and 21st galvanostatic discharge/charge curve of P-TiO_2_ and H–N-TiO_2_ electrodes at the current density of 0.1 C. The 1st discharge curve of H–N-TiO_2_ indicate a longer voltage plateau from 0.5 to 0.01 V compared to P-TiO_2_, which is matched well with the CV results and also tell us that H–N-TiO_2_ possesses a higher sodium ion storage ability. The 21st cycle galvanostatic discharge/charge curves show the similar result. According to the results mentioned above, firstly, the achieved discharge specific capacity of H–N-TiO_2_ is higher than the P-TiO_2_ electrode; secondly, for H–N-TiO_2_, the higher charge capacities could be obtained when the charge voltage is higher than 1.0 V, means that the reversible charge capacities of H–N-TiO_2_ are higher than the P-TiO_2_. In addition, Fig. 7b, c) show the galvanostatic discharge/charge curves of the electrodes at different current densities. The discharge/charge capacities of H–N-TiO_2_ are all higher than that of P-TiO_2_. And much slower performance fading of H–N-TiO_2_ is obtained at all different current stages. All these results give solid proofs of the considerable performance improvement of the H–N-TiO_2_ electrode.

## Conclusions

In summary, we developed a combination of hydrogen and nitrogen plasma assisted strategy to synthesize nitrogen doping and defect-rich TiO_2_ electrode that demonstrated an enhanced performance as anode material of sodium ion batteries. When used as anode materials, the H–N-TiO_2_ shown the best sodium storage performance, and then is H-TiO_2_, both of them demonstrated much higher specific capacities than pristine TiO_2_. The rate performances of H-TiO_2_ and H–N-TiO_2_ are significantly improved compared to P-TiO_2_. What’s more, H–N-TiO_2_ shows a 43.5% of capacity higher than the P-TiO_2_ after 400 cycles long-term discharge/charge process, and the samples show a good long cycling stability as well, the Coulombic efficiencies of all samples are nearly 99% after 50 cycles, and this could be sustained to the end of long cycling. In addition, H–N-TiO_2_ reached the stable high Coulombic efficiency earlier than the pristine material. The high-resolution images of transmission electrons microscopy shown that there is a disordered surface layer was formed after the plasma treated materials, no matter the hydrogen or nitrogen plasma process. For this improvement of the electrodes, the disordered surface layer, the oxygen vacancies and the nitrogen doping play significant roles together in enhancing the electrochemical sodium storage performance.
